# Measurement of abdominal circumference in preterm infants

**DOI:** 10.1186/s13104-015-1657-z

**Published:** 2015-11-26

**Authors:** Ilze Meldere, Valdis Urtans, Aigars Petersons, Zane Abola

**Affiliations:** Children’s Clinical University Hospital, Riga, Latvia; Pauls Stradins Clinical University Hospital, Riga, Latvia; Department of Paediatric Surgery, Riga Stradins University, Riga, Latvia

**Keywords:** Abdominal circumference, Anthropometric measurements, Premature newborn

## Abstract

**Background:**

Body weight, length and head and thoracic circumference are routinely measured in obstetric and neonatal departments. Reference values for these measurements have been established for the neonatal population. Neonatal abdominal circumference is not routinely measured, and no reference values for this measurement have been determined. To evaluate the increase in abdominal circumference in newborns with abdominal pathology such as necrotizing enterocolitis, information about normal abdominal circumference in healthy neonates shortly after birth is needed. The aim of this study was to determine the correlation between abdominal circumference and birth weight by measuring the abdominal circumference of premature neonates soon after birth.

**Methods:**

Abdominal circumference was measured within 30 min of birth in 220 neonates born between 23 and 35 weeks’ gestation.

**Results:**

There was no statistically significant difference in abdominal circumference between boys and girls in the study population. A specific formula for estimating normal abdominal circumference was developed: y = 0.0053x + 14.83 (y = abdominal circumference in cm; x = body weight in g; 0.0053 = regression coefficient; 14.83 = regression constant).

**Conclusion:**

A positive linear correlation between abdominal circumference and birth weight was found in infants at birth. The correlation can be summarized as a linear regression equation. Further studies are needed to investigate possible factors associated with abdominal circumference in fed versus unfed preterm infants.

## Background

Anthropometric measurements of infants, including body weight, length and head circumference, are routine procedures in obstetric and neonatal departments. Reference values have been determined for body weight, length and head and thoracic circumference [1]. Evaluation of abdominal circumference in neonates is not a routine procedure and reference values for this measurement have not been established. Measurement of abdominal circumference is necessary to ascertain the size of the abdominal viscera in healthy newborns and to objectively determine and specify the abdominal circumference in patients with necrotizing enterocolitis (NEC) and other diseases of the abdominal cavity. To objectively determine the increase in abdominal circumference in newborns with NEC, measurements of newborns without abdominal pathology should be ascertained soon after birth.

The aim of this study was to measure abdominal circumference in premature babies within 30 min of birth and to determine the correlation between abdominal circumference and birth weight.

## Methods

Data were obtained prospectively from premature newborns (under 36 weeks’ gestational age) born between September 2011 and December 2012 at Pauls Stradins Clinical University Hospital, Riga, Latvia. Newborns with one or more conditions that could affect body composition or abdominal circumference, such as diabetes, major congenital malformations, genetic/chromosomal abnormalities, and oedema, were excluded from the study. The Ethics Committee of Children’s Clinical University Hospital approved this study (registration number 40003457128, approved 30 August 2010). Parents or guardians provided written informed consent for participation in the study.

Abdominal circumference was determined routinely at the same time as other anthropometric measurements (weight, head circumference, chest circumference and length). We measured the abdominal circumference of 220 preterm babies (105 boys and 115 girls) within 30 min of birth.

No unified methodology exists for measuring abdominal circumference in newborns. We developed a methodological protocol for defining waist circumference by modifying the protocol for paediatric patients described in the Anthropometry Procedures Manual of the National Health and Nutrition Examination Survey and the protocol for defining neonatal waist circumference developed at the University of Wisconsin, Milwaukee [2, 3]. Our protocol involved measuring the abdominal circumference at the umbilicus 30 min after birth, at the time of body weight, length and head and thoracic circumference measurement [4–7]. We used a disposable 61.9-cm paper tape measure (D. P. Abrams, Liverpool, UK). Measurements were made after cleaning the newborn, but before breastfeeding or any other enteral feeding. Measurements were obtained with the newborn lying supine, with the tape measure placed under the back, perpendicular to the spine at the level of the umbilicus, touching skin, but not compressing the tissue [3]. Neonatal weights were measured on an infant-weighing scale, which was calibrated daily for accuracy. Physical examination of newborns and anthropometric measurements were performed in the delivery room or in the neonatal care room. Abdominal circumference was measured by two independent investigators, one of whom participated in all examinations. The values obtained by each investigator were compared and statistical reliability was determined. Data were obtained and *t*-test analysis was performed with Microsoft Excel 2010 and SPSS v. 19.0

## Results

The study population included 220 premature newborns born between September 2011 and December 2012 at Pauls Stradins Clinical University Hospital, Riga, Latvia. There were 105 boys (47.7 %) and 115 girls (52.3 %). Characteristics of the study population according to sex are shown in Table 1.

**Table 1:** Characteristics of the study population by sex

Characteristic	Boys		Girls		p value for difference	Total	
	Mean	SD	Mean	SD		Mean	SD
Total group (n = 220)							
Gestational age (weeks)	31.1	2.41	30.1	3.31	0.007	30.6	2.96
Birth weight (g)	1766.5	447.49	1599.9	560.13	0.016	1678.9	515.2
Abdominal circumference (cm)	24.1	2.66	23.5	3.36	0.101	23.8	3.05

In the overall population, abdominal circumference did not differ between boys and girls (Table 1). There was no statistically significant difference in birth weight according to sex. Gestational age ranged from 23 to 35 weeks.

There was close correlation between the abdominal measurements of the two investigators (r = 0.99).

**Fig. 1:**
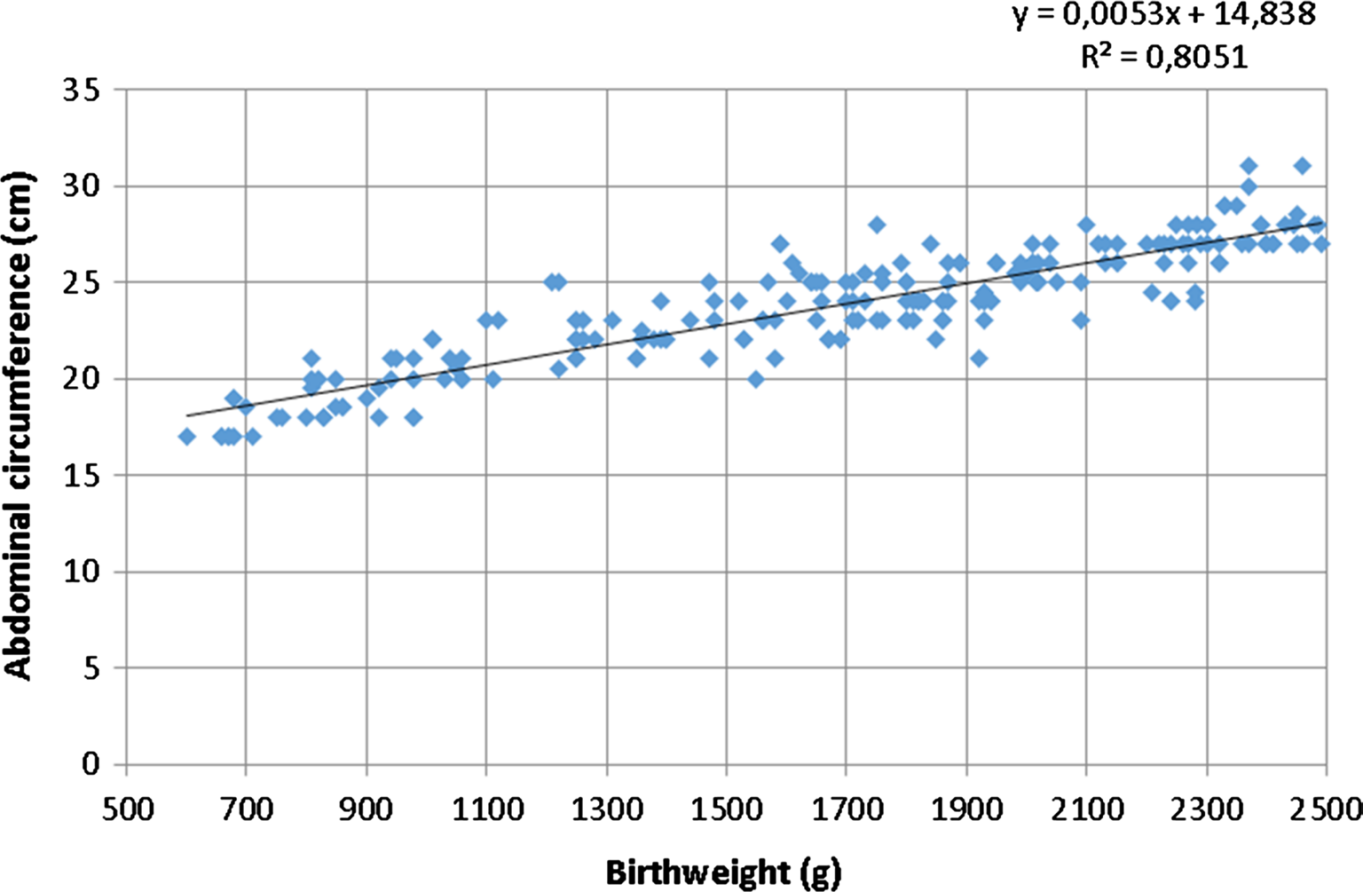
Abdominal circumference and birth weight in preterm neonates

Independent sample linear regression analysis showed that neonatal abdominal circumference had a close positive correlation with birth weight (R2 = 0.8) (Fig. 1).

The linear regression model including the independent variables sex and birth weight showed no statistically significant relationship between abdominal circumference and sex. Excluding sex from the linear regression model, there was statistically significant relationship between birth weight and abdominal circumference. The independent variable “birth weight” explained 81 % of the variation in abdominal circumference.

The linear regression equation for estimating and predicting normal abdominal circumference in unfed preterm infants is as follows: y = 0.0053x + 14.83 (y = abdominal circumference in cm; x = body weight in g; 0.0053 = regression coefficient; 14.83 = regression constant).

## Discussion

Anthropometric parameters in newborns, such as body weight, length and head and thoracic circumference have broad application during the neonatal period. These measurements can be used by general practitioners to assess a newborn’s physical development, and neonatologists use this information in the neonatal unit. These measurements are easy to interpret because there are clear standards, with available growth charts and percentile scores.

Abdominal circumference is influenced by several factors: resistance of the anterior abdominal wall, timing of feeding and defecation, phase of breathing and amount of fat. These factors might contribute to the lack of accepted standards for neonatal abdominal circumference. In this study we measured abdominal circumference in preterm neonates shortly after birth before enteral feeding to determine the average values and correlations with birth weight. Measurements of waist circumference have been widely studied. However, few studies of the relationship between abdominal circumference and birth weight have been reported, and prior studies are associated with measurement errors [8, 9]. Neonatal anthropometric parameters are assessed in many countries, including the United States, United Kingdom, Germany, India, China, New Zealand, Spain and Brazil [10–15]. Several studies have suggested that anthropometric parameters in newborns, including waist circumference, should be considered in the context of the anthropometric parameters of the mother and foetus, which allow antenatal prediction of the size of the newborn [16, 17]. The vast majority of studies on the anthropometric parameters of newborns focus on intrauterine growth retardation and discrepancies between body weights and other physical measurements and gestational age. To evaluate foetal physical development, antenatal ultrasonography is performed [17–19].

Studies on anthropometric parameters have not emphasized the importance of measuring abdominal circumference in newborns. The clinical signs of NEC, including increased abdominal circumference, have diagnostic importance. With each clinical stage of NEC, abdominal circumference increases [20, 21]. This increase can be shown with dynamic measurement of abdominal circumference, which can be achieved by routinely measuring waist circumference and other anthropometric parameters in the delivery department. This initial data can be used in future dynamic measurements of abdominal girth, if NEC or other diseases of the abdominal organs develop. No common guideline has been developed in Latvia or in other countries for abdominal circumference measurement in neonatal units. Knowledge of waist circumference measurements in all neonates and sick newborns admitted to neonatal units could be a meaningful indicator of prognosis and could aid early diagnosis of diseases of the abdominal cavity.

Neonatal abdominal circumference values obtained with the linear regression graph developed in our study can be used clinically in neonatal units, allowing more precise determination of the increase in waist circumference in patients with NEC and other diseases of the abdominal cavity.

Linear regression analysis in this study resulted in a formula that allows abdominal circumference to be calculated. The ability to calculate neonatal abdominal circumference is especially important in cases in which a premature newborn is transferred to a specialized unit with suspected gastrointestinal pathology. If the newborn’s abdominal circumference at birth is not known, a value can be calculated with the described formula and compared with measurements of abdominal circumference at different time points. This information allows practitioners to predict the risk of NEC or other gastrointestinal pathology, and to initiate early medical or surgical treatment, which can affect morbidity and mortality among premature newborns.

## Conclusions

We found no statistically significant difference in the mean abdominal circumference of boys versus girls in the study population.

There was a positive correlation between abdominal circumference and birth weight. Birth weight can be used to estimate the normal abdominal circumference in unfed preterm infants. Further studies are needed to investigate possible differences in factors related to abdominal circumference in fed versus unfed preterm infants. These data could be useful in predicting the risk of NEC and other gastrointestinal pathology.
